# Muscle ultrasound: A useful tool in newborn screening for infantile onset pompe disease

**DOI:** 10.1097/MD.0000000000008415

**Published:** 2017-11-03

**Authors:** Hsuen-En Hwang, Ting-Rong Hsu, Yueh-Hui Lee, Hsin-Kai Wang, Hong-Jen Chiou, Dau-Ming Niu

**Affiliations:** a Department of Radiology; b Department of Pediatrics, Taipei Veterans General Hospital, Taipei, Taiwan.

**Keywords:** infantile-onset glycogen storage disease (Type 2), late-onset glycogen storage disease (Type 2), muscle ultrasound, newborn screening, pseudodeficiency

## Abstract

Our study aimed to evaluate the utility of muscle ultrasound in newborn screening of infantile-onset Pompe disease (IOPD) and to establish a system of severity grading. We retrospectively selected 35 patients with initial low acid alpha-glucosidase (GAA) activity and collected data including muscle ultrasound features, GAA gene mutation, activity/performance, and pathological and laboratory findings. The echogenicity of 6 muscles (the bilateral vastus intermedius, rectus femoris, and sartorius muscles) was compared to that of epimysium on ultrasound and rated either 1 (normal), 2 (mildly increased), or 3 (obviously increased). These grades were used to divide patients into 3 groups. IOPD was present in none of the grade-1 patients, 5 of 9 grade-2 patients, and 5 of 5 grade-3 patients (*P* < .001). Comparing grade-2 plus grade-3 patients to grade-1 patients, muscle ultrasound detected IOPD with a sensitivity and specificity of 100.0% (95% confidence interval [CI]: 69.2%–100%) and 84.0% (95% CI: 63.9%–95.5%), respectively. The mean number of affected muscles was larger in grade-3 patients than in grade-2 patients (4.2 vs. 2.0, *P* = .005). Mean alanine transaminase (ALT), aspartate transaminase (AST), creatine kinase (CK), and lactate dehydrogenase (LDH) levels were differed significantly different between grade-3 and grade-1 patients (*P* < .001). Because it permits direct visualization of injured muscles, muscle ultrasound can be used to screen for IOPD. Our echogenicity grades of muscle injury also correlate well with serum levels of muscle-injury biochemical markers.

## 1 Introduction

Acid alpha-glucosidase (GAA, also called acid maltase) deficiency (Pompe disease) was the first identified lysosomal storage disease. Acid glucosidase deficiencies lead to intralysosomal accumulations of glycogen, resulting in cellular dysfunction and progressive damage to skeletal and cardiac muscle. There are 2 phenotypic forms of the disease: infantile- and late-onset. The more severe form, infantile-onset Pompe disease (IOPD), is characterized by progressive cardiac hypertrophy, hypotonia, and respiratory distress. IOPD is consistently fatal without treatment. However, late-onset Pompe disease (LOPD) has a wide spectrum of clinical presentations characterized by myopathy and respiratory insufficiency, although the cardiac muscle is usually not involved.^[1–3]^

In 2006, enzyme replacement therapy was approved for the treatment of IOPD, and its initiation early in the course of symptom development might be the key to better outcomes for patients with IOPD.^[2–7]^ In Taiwan, the use of a fluorescence assay to measure GAA activity in dried blood spots (DBS) to screen newborns remains problematic as the test does not adequately differentiate IOPD patients from LOPD patients or false-positive cases with pseudodeficiency mutation. Development of new screening procedures has thus become an urgent priority.^[4,8–11]^

Muscle ultrasound is an inexpensive, established, highly available, painless, and non-sedation-requiring imaging method for the screening of children with suspected muscle disease and can also be arranged simultaneously with muscle biopsy for pathological confirmation.^[12–17]^ Previous studies of both IOPD and LOPD have focused on the correlation of muscle ultrasound findings to clinical findings, and have even suggested using muscle ultrasound as a screening method for subclinical LOPD.^[18,19]^ The purpose of our study was to evaluate muscle ultrasound as an alternative method for newborns with initial low GAA activity and to establish a suitable system of grading muscle damage as a correlate to changes in clinical parameters.

## 2 Material and methods

### 2.1 Study population and study design

**Figure 1:**
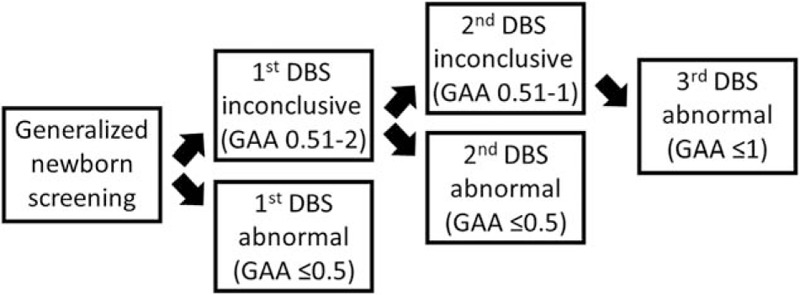
Flow chart showing the screening procedure for identifying patients with low GAA level (μmole/L/hr) in our hospital. DBS = dried blood spots, GAA = acid alpha- glucosidase.

This retrospective, observational study selected 43 patients consecutively with initial low GAA activity (as detected by the DBS test) who also had a muscle ultrasound examination during or after 2013 at our hospital. The screening algorithm is shown (Fig. 1). We excluded 8 patients with disease onset who were receiving enzyme replacement therapy at the time of their ultrasound examination and included the remaining 35 patients (10 patients who were newborn and confirmed to have IOPD and a control group consisting of 25 patients who were diagnosed with either LOPD or pseudodeficiency). The pediatric geneticist decided that none of the patients in the control group needed to receive enzyme therapy at the time of their ultrasound examinations. GAA gene mutation, activity/performance, and pathological and laboratory data were collected. The laboratory tests measured serum levels of alanine transaminase (ALT), aspartate transaminase (AST), creatine kinase (CK), and lactate dehydrogenase (LDH).

Several ultrasound machines in our radiology department were used to perform muscle ultrasound including: an Aixplorer (Supersonic Imagine SA, Aix-en Provence, France) using an SL 15–4 linear transducer (51 mm footprint with an effective bandwidth of 4–15 MHz); S2000 (Siemens-Acuson, Mountainview, CA) using a 14-L5 linear transducer (39 mm footprint with an effective bandwidth of 5–14 MHz); S3000 (Siemens-Acuson) using a 9L4 linear transducer (40 mm footprint with an effective bandwidth of 4–9 MHz); and LOGIQ E9 (GE, Wauwatosa, WI) using an ML6–15 linear transducer (58 mm footprint with an effective bandwidth of 4–15 MHz). The frequency, time gain compensation, overall gain, depth, and focus settings were not fixed but adjusted individually.

The reporting in our study followed the “Strengthening the Reporting of Observational Studies in Epidemiology (STROBE) Statement”.^[20]^ The protocol of our study was approved by the institutional review board of our hospital (approval number: 2014–09–004B), and informed consent to participate in the study was obtained from all patients from their legal representatives.

### 2.2 Outcome measures

**Figure 2:**
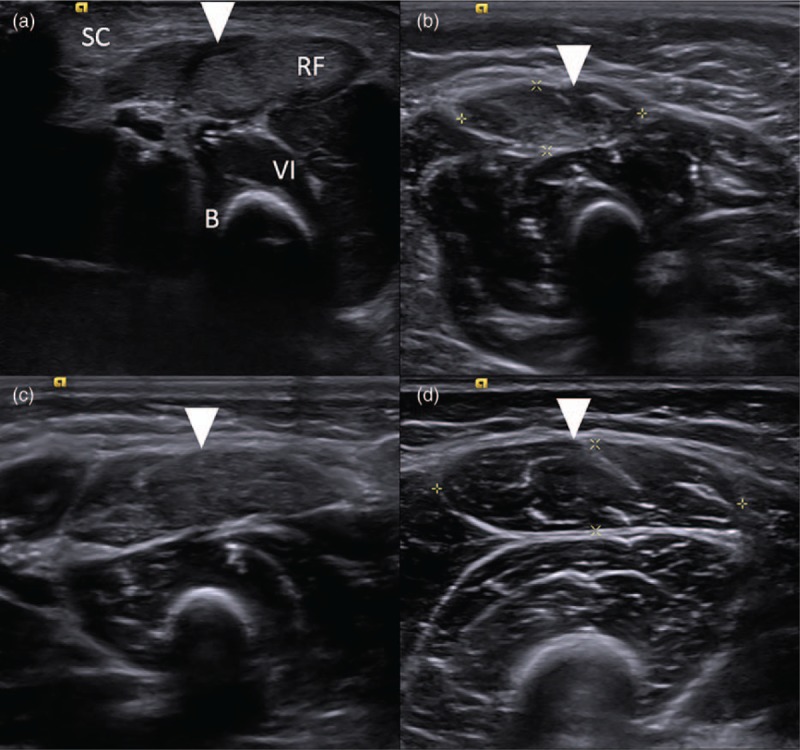
Representative muscle ultrasound images of the thigh with arrowheads pointing to the rectus femoris muscle in an (A) 10-day-old boy with infantile-onset Pompe disease classified as grade 3; (B) 46-day-old girl with infantile-onset Pompe disease classified as grade 2; (C) 30-day-old girl with pseudodeficiency mutation classified as grade 2; (D) 5-year-old girl identified as having late-onset Pompe disease by molecular genetic testing and grade 1 disease by muscle ultrasound examination. B = bone reflection, RF = rectus femoris muscle, SC = subcutaneous fat, VI = vastus intermedius muscle.

Two radiologists specializing in musculoskeletal imaging reviewed the images of these 35 patients, including the bilateral sartorius, rectus femoris, and vastus intermedius muscles. We used a modified Heckmatt rating scale to divide muscle echogenicity (relative to that of its perimysium and epimysium) into 3 grades: 1 = normal, with no increase in muscle echogenicity compared to its epimysium and clear appearance of the perimysium; 2 = mild (<50%) increase in muscle echogenicity compared to its superficial epimysium with still definable perimysium; 3 = obvious (>50%) increase in muscle echogenicity compared to its superficial epimysium with indistinct perimysium.^[21]^ At least 2 cross-sectional images of each thigh were acquired. The number of affected muscles was recorded for each patient (Fig. 2).

Muscle biopsy was performed in 13 patients for pathological examination (10 in the IOPD group and 3 in the control group) when the diagnosis of IOPD was highly suspected by clinical presentation. Under sonographic guidance using the trocar method, a 14-G 11-cm biopsy needle was inserted into the targeted muscle and the specimen was collected, stored frozen on dry ice, and sent out for pathological examination. No hematoma or other biopsy complication developed in any patient.

### 2.3 Statistical analysis

Statistical analysis was performed using Microsoft Excel 2010 and IBM SPSS 22. Differences between echogenicity groups were assessed using the *χ*^2^ test, Mann–Whitney test, and analysis of variance (ANOVA) with Bonferroni test, as appropriate. Descriptive statistics and Shapiro-Wilk tests were also performed before analysis.

## 3 Results

### 3.1 Description of the study population

For the IOPD group, the average interval between birth and the muscle ultrasound procedure was 16.5 days (range 10–47 days). Ultrasound-guided muscle biopsies were performed on the same day in 9 patients and the next day in the remaining 1 patient. The pathological reports showed glycogenesis by electron microscopic examination in all 10 IOPD patients. The average interval between laboratory analysis and muscle ultrasound was 5.5 days.

With regard to the control group, muscle ultrasound was not included in our routine screening program, so the mean time interval between birth and muscle ultrasound was extended to 30.3 months (range 9 days–6 years). Only 3 patients in the control group received ultrasound-guided muscle biopsies; however, none of them were diagnosed with biopsy-proven IOPD. The average interval between laboratory analysis and muscle ultrasound was 13.8 days.

The characteristics and descriptive statistics of the IOPD group and control group are listed in Tables 1 and 2.

**Table 1:**
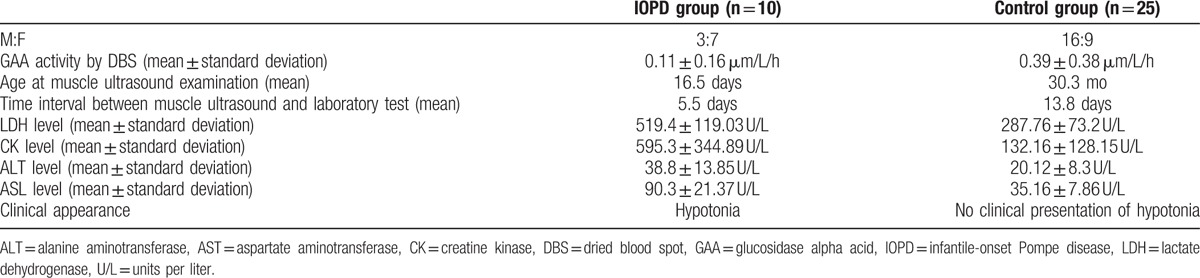
Population features.

**Table 2:**
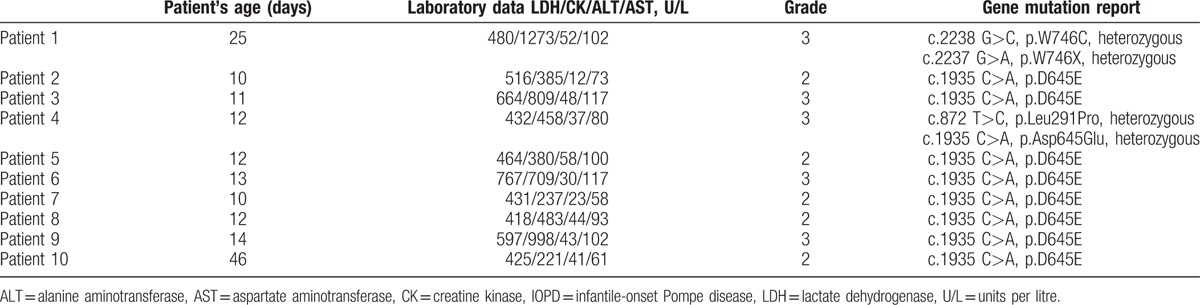
IOPD patients’ features.

### 3.2 Muscle ultrasound findings

IOPD was diagnosed in none of the 21 grade-1 patients (0%), 5 out of 9 of the grade-2 patients (55.6%), and all 5 of the grade-3 patients (100%). The *χ*^2^ test revealed a statistically significant association between muscle injury grade and the presence of IOPD (*P* < .001).

Using our possible screening tool, we could differentiate patients with grade-2 or grade-3 disease from those with grade-1 disease with a sensitivity of 100.0% (95% confidence interval [CI]: 69.2%–100%) and specificity of 84.0% (95% CI: 63.9%–95.5%) (Table 3).

**Table 3:**
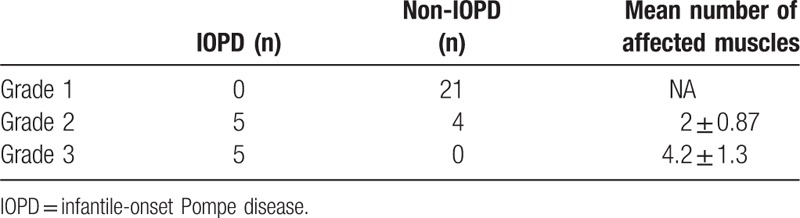
Muscle ultrasound classification.

The Mann–Whitney test revealed that the mean number of affected muscles was significantly larger in grade-3 patients than grade-2 patients (4.2 and 2.0, *P* = .005).

### 3.3 Correlation between muscle ultrasound findings and laboratory data

**Figure 3:**
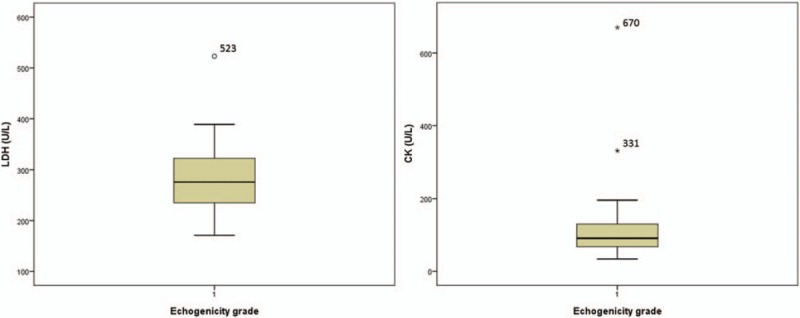
Box plots for the LDH and CK levels in the grade-1 group; the rectangle reveals the lower and upper quartiles, and a vertical line inside indicates the median value. The outlier is defined as upper quartiles ± 3 × interquartile range. Two patients were therefore excluded from the grade-1 group because of LDH and CK outlier data. CK = creatine kinase, LDH = lactate dehydrogenase.

Since our intent was to test the relationship of 4 different laboratory parameters to muscle ultrasound findings, we excluded 2 grade-1 patients with outlier data identified by preliminary descriptive statistics and normality analysis from the analysis set (Fig. 3).

**Figure 4:**
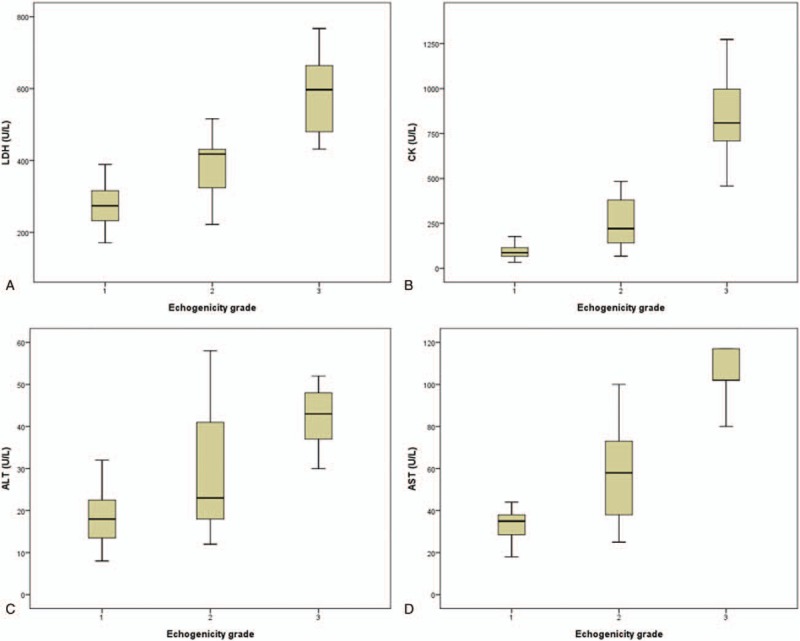
Box plots for (A) LDH, (B) CK, (C) ALT, and (D) AST levels in different groups divided according to echogenicity grade. ALT = alanine aminotransferase, AST = aspartate aminotransferase, CK = creatine kinase, LDH = lactate dehydrogenase.

ANOVA demonstrated a significant difference in mean laboratory values between patient groups, with the highest lab values in grade-3 patients, and the lowest values in grade-1 patients (*P* < .001) (Table 4). The results are also shown as box plots (Fig. 4).

**Table 4:**
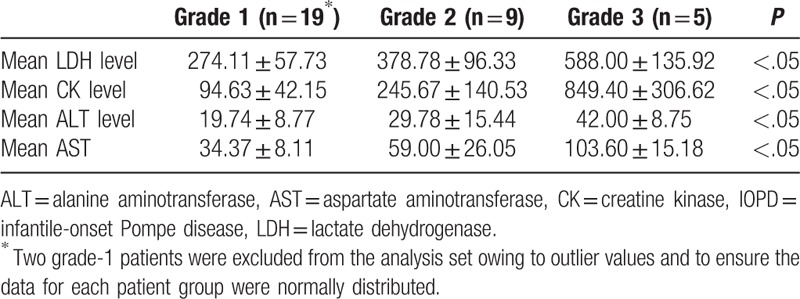
Comparison of groups classified by muscle ultrasound grade.

The Bonferroni test revealed significant differences in levels of all 4 laboratory variables between grade-1 and grade-3 patients (*P* < 0.001); CK and LDH levels between grade-1 and grade-2 patients (*P* = .032 and .012 respectively); and CK, AST, and LDH levels between grade-2 and grade-3 patients (*P* < .001).

## 4 Discussion

Our study is the first to demonstrate that muscle ultrasound findings and serum levels of laboratory parameters are correlated early in the development of IOPD, even before enzyme replacement therapy is initiated. Instead of choosing neonates without neuromuscular disease, we selected LOPD/pseudodeficiency patients to be our control group, as patients with initial low GAA activity level in newborn screening were our population of interest.

Muscle ultrasound is an established diagnostic imaging method for the screening of patients with suspected muscle diseases or neuromuscular disorders. Characteristic ultrasound findings of such diseases include increase in muscle echogenicity, atrophic change of the muscle, and loss of the underlying bone echo. Numerous studies have been conducted to evaluate the usefulness of quantitative and qualitative methods of muscle echogenicity measurement in diagnosis of muscle disease of different etiologies.^[14–17,22,23]^

In our study, patients were divided into 3 groups based on echogenecity in muscle ultrasound images, and the images were negative if graded <2, and positive if graded ≥2. The sensitivity, specificity, and positive predictive value of the test were 100%, 85.2%, and 71.4%, respectively. This result is in contrast to previous findings published in 2014 that indicated the unsuitability of ultrasound as a screening method for IOPD. However, we may ascribe this poor correlation of ultrasound score and clinical severity to the long interval between birth and the postnatal ultrasound examination (average 16.25 months), small sample size (4 cases), or differences in disease response to enzyme replacement therapy.^[18]^ Our present results suggest that muscle ultrasound can detect IOPD in newborns with high sensitivity and specificity, and therefore that it is a useful screening tool. Moreover, the muscle biopsy reports in all the IOPD patients, which revealed glycogenosis under electron microscopic examination, also supported the relationship between the disease characteristics and muscle ultrasound findings.^[24,25]^

The biochemical parameters CK, AST, ALT, and LDH in serum have always been considered to be reliable measures of the extent of muscle injury. In previous studies, elevated serum CK, ALT, AST, and LDH levels were shown to differentiate IOPD from suspected LOPD or false positive cases with pseudodeficiency mutation.^[7,26,27]^ Our results also demonstrated a significant positive correlation between muscle ultrasound score and levels of these laboratory markers. The higher the level of these markers, the greater the increase in echogenicity of the examined muscle. Therefore, we suggest that the change in echogenicity in IOPD patients can be used to estimate disease severity, as previously found in LOPD patients.^[19]^

Two patients were outliers and excluded from the grade-1 group dataset before proceeding with the ANOVA. CK/ALT/AST/LDH levels were 331/23/42/308 and 670/15/41/523 in the first and second patients, respectively. Both patients had obvious elevation in CK and LDH levels, skewing the range away from a normal distribution in the grade-1 group. The annual follow-up laboratory evaluation revealed a subsequent decrease in CK and LDH levels in both patients to the normal range in the absence of any specific treatment. To our knowledge, serum concentrations can be increased for reasons other than muscle disease, such as exercise, muscle trauma, motor neuron disease, and renal disease.^[27–29]^ As a result, caution is warranted when diagnosing muscle injury on the basis of elevated laboratory indices alone.

In the study by Yang et al in 2013,^[7]^ laboratory indices including LDH, CK, AST, and ALT levels for 6 patients with IOPD all increased over time despite enzyme treatment. These increases may have resulted from increased physical activity or sustained skeletal myocyte damage caused by accumulation of glycogen in lysosomes. In this regard, direct visualization and grading by musculoskeletal ultrasound might have an advantage over laboratory testing in evaluating muscle damage for making long-term follow-up comparisons.

Previous studies have used grading systems, such as the Muscle Research Council (MRC) scale, the Pompe Pediatric Evaluation of Disability Inventory (PEDI) score, or the quantitative measurement system of the Cooperative International Neuromuscular Research Group^[18,19,30]^ to assess the clinical condition of multiple muscles. In contrast, our study evaluated only the bilateral vastus intermedius, rectus femoris, and sartorius muscles since lower-extremity hypotonia is usually the very first clinical presentation observed in IOPD patients. Although our study found that the mean number of affected muscles was significantly larger in the grade 3 group than grade 2 group, further studies will be required to elucidate the correlation between disease severity and the extent of muscle damage in more and different muscles.

Our study had several limitations that warrant mention. The retrospective nature of our study resulted in a control group that was older than the IOPD group. Moreover, the small sample size might not be representative enough to generalize the findings. In addition, qualitative grading based on visual inspection is subjective and can hamper the reliability of the result. The echogenicity was not quantitatively measured because several different ultrasound machines were used and the machine settings were not fixed but adjusted individually in different machines and no correction for differences in reliability between systems could be made. Thus, a complete prospective study including healthy controls using a single machine with consistent settings for quantitative analysis and even ultrasound elastography analysis would be helpful.

## 5 Conclusion

Our study showed that muscle ultrasound can be a useful muscle injury visualization tool for screening newborns for IOPD. The grading used in our study was also well correlated with serum levels of biochemical indicators of muscle injury.
